# Evidence of Prognostic Relevant Expression Profiles of Heat-Shock Proteins and Glucose-Regulated Proteins in Oesophageal Adenocarcinomas

**DOI:** 10.1371/journal.pone.0041420

**Published:** 2012-07-24

**Authors:** Julia Slotta-Huspenina, Daniela Berg, Karina Bauer, Claudia Wolff, Katharina Malinowsky, Lukas Bauer, Enken Drecoll, Marcus Bettstetter, Marcus Feith, Axel Walch, Heinz Höfler, Karl-Friedrich Becker, Rupert Langer

**Affiliations:** 1 Institute of Pathology, Technische Universität München, Munich, Germany; 2 Department of Surgery, Technische Universität München, Munich, Germany; 3 Institute of Pathology, Helmholtz-Zentrum München, Oberschleissheim, Germany; 4 Teilgemeinschaftspraxis Molekularpathologie, München, Germany; 5 Institute of Pathology, University of Bern, Bern, Switzerland; Boston University Medical School, United States of America

## Abstract

A high percentage of oesophageal adenocarcinomas show an aggressive clinical behaviour with a significant resistance to chemotherapy. Heat-shock proteins (HSPs) and glucose-regulated proteins (GRPs) are molecular chaperones that play an important role in tumour biology. Recently, novel therapeutic approaches targeting HSP90/GRP94 have been introduced for treating cancer. We performed a comprehensive investigation of HSP and GRP expression including HSP27, phosphorylated (p)-HSP27^(Ser15)^, p-HSP27^(Ser78)^, p-HSP27^(Ser82)^, HSP60, HSP70, HSP90, GRP78 and GRP94 in 92 primary resected oesophageal adenocarcinomas by using reverse phase protein arrays (RPPA), immunohistochemistry (IHC) and real-time quantitative RT-PCR (qPCR). Results were correlated with pathologic features and survival. HSP/GRP protein and mRNA expression was detected in all tumours at various levels. Unsupervised hierarchical clustering showed two distinct groups of tumours with specific protein expression patterns: The hallmark of the first group was a high expression of p-HSP27^(Ser15, Ser78, Ser82)^ and low expression of GRP78, GRP94 and HSP60. The second group showed the inverse pattern with low p-HSP27 and high GRP78, GRP94 and HSP60 expression. The clinical outcome for patients from the first group was significantly improved compared to patients from the second group, both in univariate analysis (p = 0.015) and multivariate analysis (p = 0.029). Interestingly, these two groups could not be distinguished by immunohistochemistry or qPCR analysis. In summary**,** two distinct and prognostic relevant HSP/GRP protein expression patterns in adenocarcinomas of the oesophagus were detected by RPPA. Our approach may be helpful for identifying candidates for specific HSP/GRP-targeted therapies.

## Introduction

Oesophageal adenocarcinoma arising from precancerous Barrett’s Metaplasia (Barrett’s Carcinoma) is a very aggressive tumour and is often diagnosed only at an advanced stage. Despite advances in surgery and the introduction of neoadjuvant treatment of locally advanced carcinomas, the prognosis for invasive oesophageal adenocarcinoma is poor, with a five-year survival rate of less than 50% [1], [2]. This may also be due to the considerable rate of chemotherapy resistance in these tumours [3]–[6]. For that reason, it is of major importance to develop individualised therapeutic concepts and alternative therapeutic strategies beyond conventional chemotherapeutic or surgical treatment to improve the current treatment of patients with this disease.

Although there is increasing knowledge about the molecular background of this disease [7], to date, no valid biomarkers have been identified to predict prognosis or chemotherapy response. Previously, we showed that the regulation and expression of several molecular chaperones such as heat-shock proteins (HSPs) and glucose-regulated proteins (GRPs) may have an important impact on the tumour biology of oesophageal adenocarcinomas with respect to prognosis and chemotherapy response [8], [9]. Moreover, novel therapeutic approaches targeting HSPs and GRPs have been studied in preclinical models and in part introduced for cancer treatment [10]–[14]. However most data about HSP and GRP expression have been generated from smaller sample collections and/or focussed on selected proteins. Given the complexity of regulation of molecular chaperones it is likely that the biological behaviour of tumours may be influenced by the interaction and crosstalk between these molecules rather than by alterations of one single protein. In view of the presumed prognostic significance of HSPs and GRPs, we now performed a comprehensive expression analysis of the heat-shock proteins HSP60, HSP70, HSP90 and HSP27 (including its phosphorylated forms p-HSP27^(Ser15)^, p-HSP27^(Ser78)^, p-HSP27^(Ser82)^) and the glucose-regulated proteins GRP 94 and GRP78 in a well-characterized collection of primary resected oesophageal adenocarcinomas from patients who received neither neo-adjuvant nor adjuvant treatments. We analysed the expression of these proteins using reverse phase protein array (RPPA) technology, which also allows quantitative analysis of protein expression from formalin-fixed, paraffin-embedded (FFPE) tissue [15], [16]. Additionally, immunohistochemistry and real-time quantitative RT-PCR (qPCR) were carried out. The results of the expression studies were correlated with pathological features and patients’ clinical outcome.

## Materials and Methods

### Ethics Statement

All patients gave informed written consent, and the study was approved by the Ethics Committee of the Technische Universität München, Munich, Germany (#2056/08).

### Patient Characteristics and Tissue Samples

Formalin-fixed, paraffin-embedded (FFPE) tumour samples from 92 patients with oesophageal adenocarcinomas, who were treated between 1991 and 2006 in the department of surgery of the Klinikum Rechts der Isar der Technischen Universität München, Germany, were investigated. Primary resection of the tumours was conducted by trans-thoracic or trans-hiatal oesophagectomy without (neo-)adjuvant chemotherapy or radiochemotherapy. The resection specimens were processed immediately after surgery i.e. they were opened by a pathologist and fixed in 4.5% buffered formalin for 24–48 hours. Patient and tumour characteristics are given in **Table 1**. The median age of the patients was 62 (range 33–82). Of the 92 patients included in this study, 51 patients died of disease, with a median overall survival (OS) of 33.2 months (95% CI = 17.9–48.5) and a median disease-free survival (DFS) of 31 months (95% CI = 22.7–39.3).

**Table 1:** Clinico-pathologic characteristics of the patients.

Factor		Number of patients	%
		92	
***Gender***			
Male		78	84.8
female		15	16.3
***UICC pT catergory***			
pT1		30	32.6
pT2		21	22.8
pT3		41	44.6
***UICC pN category***			
pN0		46	50
pN1/2		46	50
***Metastases***			
cM0		84	91.3
cM1		8	8.7
***Tumour grading***			
G1		2	2.2
G2		39	42.4
G3		51	55.4
***Resection status***			
R0		76	82.6
R1		16	17.4
***Survival***			
alive		41	44.6
dead of disease		51	55.4

pT: tumour status as determined in pathology; pN: lymph node status as determined in pathology; cM: occurrence of distant metastases as determined by attending physician, cM0: no distant metastases, cM1: distant metastases present.

### Protein Extraction and Antibodies

Protein extraction was performed as previously described [16]. Briefly, FFPE tumour tissue from three 10 µm sections was deparaffinized (xylene for two times 10 min) and rehydrated (100%/90%/70% ethanol for five min each). The tumour tissue was microdissected, and approximately 0.5 cm^2^ tissue from three 10 µm-thick sections was processed in 100 µl of extraction buffer (EXB Plus, Qiagen GmbH, Hilden, Germany). Haemorrhagic or necrotic areas were excluded to obtain a percentage of tumour tissue of at least 80%. Protein concentrations were determined using the Bradford protein assay according to the manufacturer’s instructions (BioRad, Hercules, CA). Randomly selected lysates were probed for β-actin by Western blot to verify the success of the protein extraction and the suitability of the material for RPPA analysis. All protein lysates that were analysed showed a clear β-actin band by Western blot. Before performing RPPA for HSP/GRP expression analysis, all antibodies were validated by Western blot using proteins extracted from formalin-fixed tissues. A detailed list of the antibodies used is given in **Table 2**.

**Table 2:** Antibodies used for immunohistochemistry (IHC) and for Western blot (WB)/RPPA analysis.

			IHC	WB/RPPA
Protein	Antibody	Distributor	Dilution	
HSP27	#2402	Cell signalling, Danvers, USA	1∶250	1∶1000
Phospho-HSP^(Ser15)^	#ab39399	Abcam, Cambridge, UK	1∶500	1∶1000
Phospho-HSP^(Ser78)^	#2405	Cell signalling, Danvers, USA	1∶500	1∶1000
Phospho-HSP^(Ser82)^	#2401	Cell signalling, Danvers, USA	1∶100	1∶1000
HSP60	#ab46798	Abcam, Cambridge, UK	1∶2000	1∶2000
HSP70	#ab17850	Abcam, Cambridge, UK	1∶1	1∶50
HSP90	#ab1429	Abcam, Cambridge, UK	1∶100	1∶200
GRP78	#ab32618	Abcam, Cambridge, UK	1∶1000	1∶1000
GRP94	#sc1794	Santa Cruz Biotechnology Inc., CA, USA	1∶5000	1∶500

### Reverse Phase Protein Arrays (RPPA)

RPPAs were generated using the Calligrapher MiniArrayer (BioRad, Hercules, CA) in accordance with the manufacturer’s instructions [17], [18]. For every lysate and every dilution (undiluted, 1∶2, 1∶4, 1∶8, 1∶16, buffer), three replicates were applied to a nitrocellulose-coated glass slide (Grace Bio-Labs, Bend, OR) to obtain a total of 18 data points per sample. Peroxidase blocking was performed in accordance with the manufacturer’s instructions (Dako, Glostrup, Denmark). Immunodetection was performed similar to a Western blot, as previously described [19]. For the estimation of the total protein amount, arrays were stained in parallel with Sypro Ruby Protein Blot Stain (Molecular Probes, Eugene, OR) in accordance with the manufacturer’s instructions.

### Quantitative Protein Analysis

The TIFF images for the antibody-stained slides and Sypro Ruby-stained slides were analysed with MicroVigene 3.5.0.0 software (VigeneTech, Carlisle, MA). The MicroVigene signal-intensity points (MVS) were calculated by the integral of a logistic four-point fit model, which was matched optimally to the 18 data points that were obtained.

### Immunohistochemistry

Immunohistochemical staining was performed using tissue microarrays, which were constructed for the purpose of this study and contained samples from all 92 tumours. Antigen retrieval was performed in accordance with the manufacturer’s recommendations. After deparaffination and rehydration, heat-induced antigen retrieval was performed using 10 mM citrate buffer, pH 6. Subsequent to H_2_O_2_ blocking using 3% H_2_O_2_ in aqua dest. and Avidin Biotin blocking (Avidin/Biotin blocking kit; Vector Laboratories, Inc., Burlingame, CA, USA), the sections were incubated with antibodies for HSPs, followed by a secondary biotinylated antibody. Immunodetection was performed using the Dako REAL™ Detection system Peroxidase/DAB + kit (DAKO, Glostrup, DK). A detailed list of the antibodies and the dilutions is presented in **Table 2**.

The evaluation of immunohistochemical staining was performed at least by three independent observers (KB, JSH, ED). Differences were discussed at a double-header microscope to gain a final consensus. The expression was assessed based on the intensity of cytoplasmatic immunostaining and the percentage of stained tumour cells. The intensity was scored as 0 (negative), 1 (weak staining), 2 (moderate staining) or 3 (strong staining). The percentage of positive tumour cells was scored as 0 (none), 1 (<10%), 2 (10–50%), 3 (51–80%) or 4 (>80%). Multiplication of the scores for intensity and percentage resulted in a semiquantitative immunoreactive score ranging from 0 to 12. Examples of immunohistochemical staining are shown in the **File S1.** For GRP94 and GRP78, the staining results from a previously published study were used [20].

### Real-Time Quantitative RT-PCR (qPCR)

Microdissection, RNA extraction and cDNA synthesis were performed as described previously with minor modifications [21]. After tissue preparation, as described above, the microdissected tumour tissue was transferred into a sterile 1.5-ml tube containing RNA lysis buffer. Lysis was carried out at 60°C for 24 hours until the tissue was completely solubilised. RNA was purified by phenol and chloroform extraction followed by precipitation with an equal volume of isopropanol in the presence of 20 µl of 2 mol/L sodium acetate (pH 4.0) and 2 µl of 10 mg/ml glycogen at −20°C. The RNA pellet was washed once in 70% ethanol, dried and resuspended in 20 µl of RNase-free water. One microgram of RNA was transcribed into cDNA using Superscript II reverse transcriptase (Invitrogen) and 250 ng of random hexamers (Roche Diagnostics, Penzberg, Germany) in accordance with the manufacturer’s recommendations, in a final volume of 20 µl. Gene expression was quantified using RealTime ready single assays (Roche Diagnostics) for the target genes *GRP94* (ID 100489), *HSP27* (ID 100497), *HSP70* (ID 110730), *GRP78* (ID 110805), *HSP90* (ID 138013) and *HSP60* (ID 137175), and the housekeeping genes *PPIA* (ID 102088), *ALAS1* (ID 102108) and *ACTB* (ID 101125). Housekeeping genes were selected in a previous study using the RealTime ready Reference Gene Panel (Roche Diagnostics), which contains 19 different reference genes to facilitate the identification of the most suitable genes from eight different carcinoma samples. Using the GeNorm software, the reference genes *PPIA*, *ALAS1* and *ACTB* were shown to be most stably expressed in all tissues analysed. qPCR was performed in triplicate with the LightCycler 480 Instrument using LightCycler 480 Probes Master (Roche Diagnostics) and 10 ng of cDNA per well. Thermal cycler conditions comprised 45 cycles at 95°C for 10 s, 60°C for 30 s, and 72°C for 1 s. Relative mRNA expression was calculated by the ΔΔCt method using the LightCycler 480 Software with an efficiency-corrected algorithm with standard curves and triple normalization to *PPIA*, *ALAS1* and *ACTB* as reference genes.

### Statistical Analysis

Unsupervised hierarchical clustering was performed using Cluster and TreeView software. Following log transformation and centre to median calculations, average hierarchical clustering was performed using the Spearman rank correlation [22]. SPSS statistical software (IBM SPSS statistics 19) was used for additional statistical analysis. Associations between groups of patients were given in cross tabs and differences were determined using the χ^2^-test. Comparisons between groups were performed using non-parametric tests (Mann-Whitney U test; Kruskal Wallis test, Spearman rank correlation). Survival was calculated from the day of surgery. Patients with incomplete tumour resection (R1), the presence of distant metastases (cM1) at the time of surgery, or death within the first 30 days after surgery were excluded from the survival analysis that was performed using Kaplan-Meier estimates, log-rank tests and Cox’s proportional hazards regression analysis. All tests were two-sided, and the significance level was set at five %.

## Results

### Quantitative Protein Expression of HSPs and GRPs by RPPA

**Figure 1:**
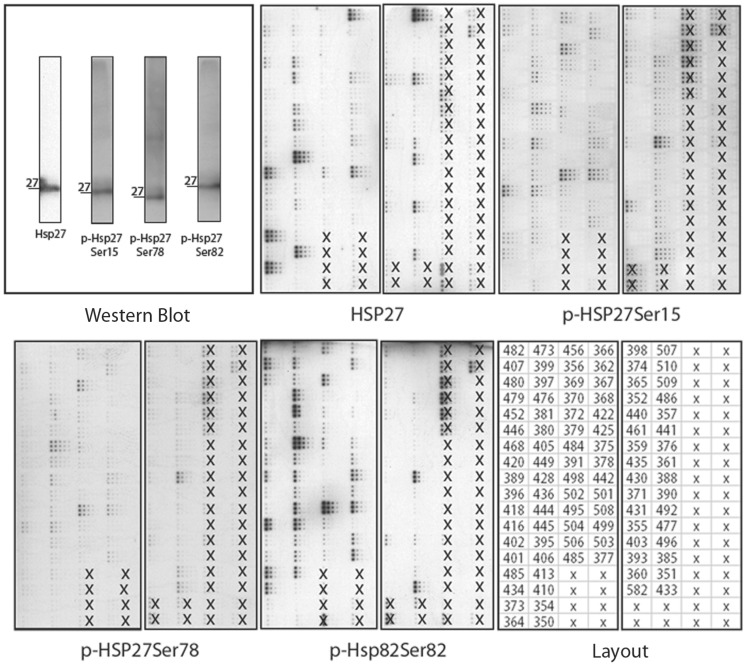
Illustration of Reverse Phase Protein Arrays (RPPA). As an example to illustrate the concept of RPPA, the results of four arrays probed with antibodies against HSP27 and its phosphorylated forms (Ser78, Ser82, Ser15) are shown. Tumour samples on the arrays which were not included in the study are marked with an “x”. Additionally, Western blots for these four antibodies are shown, to demonstrate their reliability. These images are shown for clarification; the signals must not be compared directly, as they are not normalised to the total protein content of the sample.

RPPA analysis could be carried out for 87 tumour samples; for five cases, there was not enough material for RPPA. Representative RPPA results for the unphosphorylated and the phosphorylated forms of HSP27 are given in **Figure 1**. Median quantitative protein expression (protein/SyproRuby*1000) was as follows: HSP27∶725 (range 0–6127), p-HSP27^(Ser15)^:788 (range 185–3246), p-HSP27^(Ser78)^: 850 (range 215–3671), p-HSP27^(Ser82)^: 793 (range 0–4307), HSP60∶866 (range 112–3232), HSP70∶744 (range 151–5292), HSP90∶796 (range 206–3130), GRP94∶818 range (0–4222) and GRP78∶857 (range 174–3240). We first analysed the expression levels of single proteins, and found no correlation between any of the HSPs or GRPs and pathologic parameters (UICC pTNM classification, tumour differentiation) (**File S2**). Moreover, survival analysis for the single markers revealed no significant association of any protein with overall or disease-free survival, except for a trend in association between very high p-HSP27^(Ser15)^ levels (4^th^ quartile) and better overall and disease-free survival (p = 0.073 and p = 0.11, respectively).

**Figure 2:**
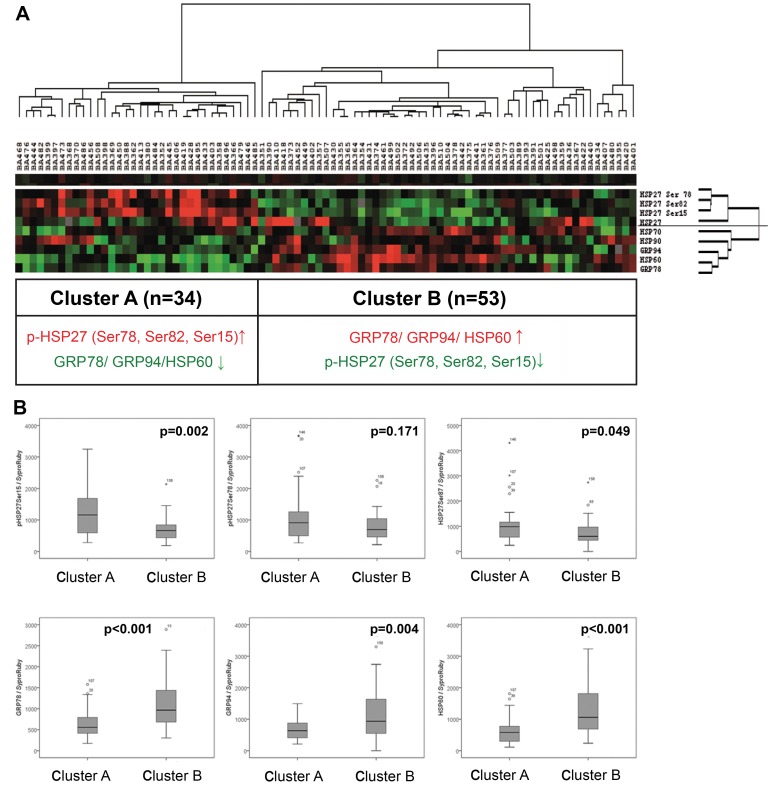
Hierarchical cluster analysis of oesophageal adenocarcinomas according to the protein expression of HSP and GRP measured by RPPA. Unsupervised hierarchical cluster analysis of 87 oesophageal adenocarcinomas based on the expression of HSP27, HSP60, HSP70, HSP90, the phosphorylated forms of HSP27 (Ser78, Ser82, Ser15), GRP 78 and GRP94, as analysed by RPPA, identified two clusters (Clusters A and B) with specific protein profiles (A). The hallmark of Cluster A is high expression of the phosphorylated HSP27 (p-HSP27) and the low expression of HSP78, GRP94 and HSP60. The hallmark of Cluster B is the inverse of Cluster A. Cluster colour key: Red – up-regulated; green – down-regulated; black – unchanged; grey – missing. Boxplots (B) illustrating the different protein expression levels assessed by RPPA analysis in tumours from Clusters A and B, as identified by hierarchical cluster analysis.

We next performed unsupervised hierarchical clustering to generate a tumour-specific protein expression pattern (**Figure 2A**). Two clusters (Clusters A and B) of the 87 patients analysed could be distinguished by their protein expression patterns: Cluster A (n = 34) consisted of carcinomas that showed a high abundance of HSP 27 and its phosphorylated forms (abbreviated p-HSP27) and low expression of GRP78, GRP94, HSP60, HSP70 and HSP90. Cluster B (n = 53) consisted of carcinomas with the inverse expression pattern (low (p-)HSP 27 and high GRP78, GRP94, HSP60, HSP70 and HSP90 expression) (**Figure 2B**).

**Figure 3:**
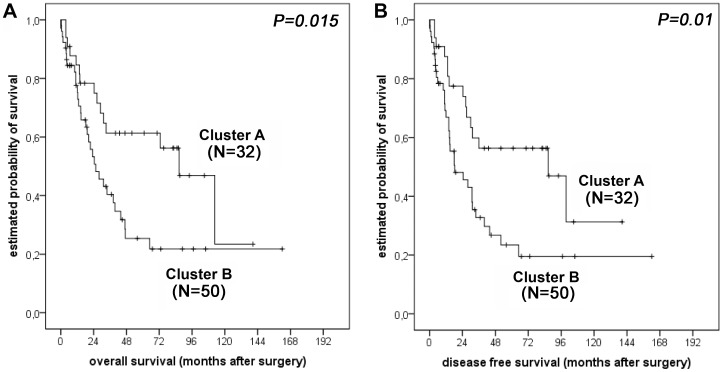
Survival analysis for oesophageal adenocarcinoma patients. Kaplan-Meier survival curves for 82 patients from Clusters A and B with respect to overall survival (A) and disease-free survival (B). Patients who died within the first month after surgery (N = 5) were excluded from the survival analysis.

To further assess the relevance of the specific HSP and GRP protein expression patterns identified, we compared the pathological features and the clinical outcome of the different patient groups. Patients from Cluster B were more likely to have lymph node metastases, although this difference was not statistically significant (p = 0.073). For the other factors (pT category, distant metastases, and tumour differentiation), no correlation was found. According to the criteria given above, survival analysis was conducted for 82 patients. Interestingly, the survival analysis showed a better prognosis for patients from Cluster A (n = 32) compared to patients from Cluster B (n = 50), with a prolonged overall survival (median OS 87 months; 95% CI = 59–114 months vs. 28 months; 95% CI = 16–40 months; p = 0.015; **Figure 3A**) and a significantly prolonged disease-free survival (median DFS 87 months; 95% CI = 30–143 months vs. 25 months; 95% CI = 9–40 months; p = 0.010; **Figure 3B**). Other factors that showed significant prognostic value in univariate analysis were: UICC pT category (OS: p<0.001; DFS: p = 0.001), presence/absence of lymph node metastases (SO and DFS: p<0.001), presence/absence of distant metastases (OS and DFS: p = 0.001), complete/incomplete tumour resection (OS: p = 0.001; DFS: p = 0.002). Multivariate analysis including UICC pT and pN category, tumour grading, and the clustered protein expression pattern showed that protein expression was the best independent prognostic indicator for overall (p = 0.029) and disease-free survival (p = 0.012) besides lymph node status (p = 0.004 and p = 0.010, respectively) (**Tables 3** **and** **4**).

**Table 3:** Multivariate Cox regression analysis (Overall survival).

Factor	Exp	95% CI for Exp(B)		p-value
		*min*	*max*	
pTcategory	1.079	0.65	1.789	0.769
lymph node status	3.569	1.507	8.454	**0.004**
distant metastases	2.148	0.828	5.569	0.116
tumour grading	1.354	0.742	2.471	0.323
Resection status	1.542	0.652	3.648	0.324
Cluster A vs. B	2.247	1.087	4.645	**0.029**

Statistically significant p-values are marked in bold letters. pT-category: pT1 vs. pT2 vs. pT3; lymph node status:absent vs. present; distant metastases: absent vs. present; tumour grading: G1 vs. G2 vs. G3; resection status: R0 vs. R1/2.

**Table 4:** Multivariate Cox regression analysis (Disease free survival).

Factor	Exp(B)	95% CI for Exp(B)		p-value
		*min*	*max*	
pTcategory	1.227	0.759	1.982	0.404
lymph node status	2.89	1.286	6.493	**0.01**
distant metastases	2.551	0.976	6.668	0.056
tumour grading	1.252	0.698	2.246	0.451
Resection status	1.227	0.531	2.836	0.633
Cluster A vs. B	2.477	1.219	5.034	**0.012**

Statistically significant p-values are marked in bold letters. pT-category: pT1 vs. pT2 vs. pT3; lymph node status:absent vs. present; distant metastases: absent vs. present; tumour grading: G1 vs. G2 vs. G3; resection status: R0 vs. R1/2.

### Immunohistochemistry

Immunohistochemical staining intensities of tumours ranged from negative (score 0) to very high (score 12) for HSP90 and from negative to high (score 8) for HSP27, p-HSP27^(Ser15)^, HSP60 and HSP70. For p-HSP27^(Ser78)^ and p-HSP27^(Ser82)^, negative vs. positive staining was assessed on the basis of single cells. Low HSP60 IHC reactivity was observed in early tumours (pT1; p = 0.009) and also in tumours without lymph node metastases (p = 0.003). For the other markers, there were no associations between pathologic parameters and IHC reactivity (see **File S3**). There was no association between IHC staining patterns and patient outcome.

### Expression Analysis of *HSP* and *GRP* mRNA

Real-time quantitative RT-PCR analysis was performed for all 92 tumours. mRNA from the target genes was detectable at various levels in cancer tissues from all patients analysed. The relative median mRNA expression levels (ratio of target gene/housekeeping genes *1000) were *HSP27*∶60 (range 10–220), *HSP60*∶2530 (650–11430), *HSP70*∶1210 (300–4510), *HSP90*∶790 (220–5110), *GRP78*∶491 (90–1660) and *GRP94*∶1080 (440–3780).

A significantly elevated level of *HSP27* mRNA expression was found in advanced tumour stages (pT2/3) as compared to early stages (pT1; p = 0.009). mRNA levels of *HSP60*, *HSP70*, *HSP90, GRP78* and *GRP94* did not correlate with pathological features (pT, pN, grade), as summarized in **File S4**. In the survival analysis, high *HSP27* mRNA levels were associated with better overall survival (p = 0.022) in univariate analysis. For the other markers, no significant association between gene expression and prognosis was evident.

### Correlation between RPPA Analysis, qPCR and Immunohistochemistry

In general, there was no strong correlation between *HSP*/*GRP* mRNA and protein expression levels, as measured either by RPPA analysis or by immunohistochemistry (**File S5)**. However, for HSP90 and HSP60, a significant correlation was found between mRNA expression levels and the immunohistochemical staining intensity (p = 0.003, p = 0.02). For HSP27, a correlation was found between mRNA expression and protein expression level as measured by RPPA analysis (p = 0.01). However, no direct correlation was found between protein expression levels detected by RPPA analysis and immunohistochemical staining results. Thus, the two patient groups identified by quantitative protein expression clustering (Clusters A and B) could not be distinguished by either qPCR or immunohistochemistry.

## Discussion

Heat-shock proteins (HSPs) and glucose-regulated proteins (GRPs) are highly conserved molecular chaperones, which are responsible for maintaining cellular integrity by promoting the correct folding of newly translated or denatured proteins. HSPs and GRPs are also referred to as stress proteins, because their expression can be up-regulated by pathological conditions such as acidosis, hypoxia or hyper- or hypothermia. HSPs and GRPs also play an important role in the regulation of apoptosis [23], [24]. In recent years, knowledge about the association of HSPs and GRPs with cancer and their important role in cancer biology has increased. Molecular chaperones have been suggested to influence tumour growth, differentiation and resistance to radio- and chemotherapy treatment, and may have a major impact on the final clinical outcome of patients with cancer [25]–[29]. For example, overexpression of HSP27 was associated with a poor clinical outcome in patients with rectal cancer [30]. Externalization of HSP70 onto the cell membrane has been shown to be tumour-specific in colon cancer cells and appears to correlate with patient prognosis [31]. In oesophageal squamous cell cancer patients, HSP70 autoantibodies were significantly increased in patients’ sera so that HSP70 has been suggested as a diagnostic marker [14]. Other studies have addressed the prognostic significance of GRP 96 and HSP90 in oesophageal squamous cell carcinomas [32]–[34].

Our group previously reported that in oesophageal adenocarcinomas, the expression of GRP78 and GRP94 varies depending on the tumour stage, with high expression levels mostly occurring either in very early or advanced stages, suggesting the complex regulation of GRPs in response to different kinds of stresses [20]. In another study, proteomic analysis followed by IHC and validation of gene expression level showed an association between high HSP27 expression and response to neoadjuvant chemotherapy in a collection of multimodal treated cases [8]. Most recently, we demonstrated that tumours with low expression of phosphorylated HSP27-family proteins and high expression of HER-family proteins were characterized by an aggressive biological behaviour [9]. These previous works focussed on selected heat shock proteins. However, the complex regulation with interactions between the various members of the HSP/GRP family are well documented [35], [36]. Therefore, identification of expression patterns of several molecules may be superior over the analysis of single markers. In order to comprehensively elucidate the role and the regulation of HSPs and GRPs in oesophageal adenocarcinomas, we studied the expression of HSP27, phosphorylated (p)-HSP27^(Ser15)^, p-HSP27^(Ser78)^, p-HSP27^(Ser82)^, HSP60, HSP70, HSP90, GRP78 and GRP94 in a well-characterized, homogenously treated collection of patients with primary resected oesophageal adenocarcinomas. We incorporated some data for HSP27 and p-HSP27, that have already been published by our group in the context of a clustering analysis with proteins from signal transduction pathways [9], but have not been presented as raw data and in combination with other HSPs and with GRPs, which is provided in the present paper. Here we show that a protein expression pattern of low p-HSP27^(Ser15, Ser78, Ser82)^ and high GRP78/GRP94/HSP60 in the tumours was significantly associated with a poorer prognosis and that this particular protein expression profile was superior to conventional pathologic prognostic factors of the UICC TNM classification system in multivariate analysis. In particular, patients who showed a high abundance of phosphorylated HSP27 protein together with low expression levels of GRP78, GRP94 and HSP60 (Cluster A) had better survival rates as compared to patients with the inverse expression pattern (Cluster B). This indicates that HSP/GRP protein expression and regulation might play a significant role in the tumour biology of oesophageal adenocarcinomas, as has already been shown in other cancer types [27], [36], [37]. Moreover, the association of a higher abundance of phosphorylated HSP27 protein with superior survival is in accordance with the observation that HSP27 phosphorylation leads to the downregulation of its chaperone activity and impairs resistance to oxidative stress [38]. The association of high expression levels of GRP78, GRP94 and HSP60 in tumours with a more aggressive clinical behaviour might lead to new therapy strategies, as therapeutic agents that inhibit HSPs and GRPs have been developed in recent years and have already been shown to act as powerful anti-tumour agents both alone and in combination with conventional chemo- or radiochemotherapy [10]–[12], [14], [39]–[41].

We have identified the prognostic, highly significant, distinct HSP/GRP-expression patterns in oesophageal adenocarcinomas by unsupervised hierarchical cluster analysis from the results of quantitative protein expression analysis using reverse phase protein array (RPPA) technology. This method has emerged as a powerful tool for the molecular characterization of cellular material. Using RPPA, it is possible to perform quantitative protein expression analysis of single proteins, to study posttranscriptional modifications such as phosphorylation or to generate signalling networks of groups of proteins. Application of RPPA for the identification of dysregulated pathways in tumour cells and for the investigation of drug response in human malignancies such as breast cancer [42], [43], lung carcinomas [44], prostate cancer [45] or non-solid neoplasms like leukemias [46] has been previously published. For the investigation of large case collections with well characterized patient data the most widely used material in cancer research represent the formalin-fixed, paraffin-embedded (FFPE) tissue collections which are stored in the pathology departments. Due to marked technical improvements the usage of this tissue now has become possible for a large range of different molecular methods. Therefore, not only robust molecular analyses like immunohistochemistry or DNA based investigations, but also more sensitive methods like microRNA or mRNA can be applied as a reliable approach in the search for biomarkers [47]. As described recently by our group [48]–[50], the highly promising RPPA technology is also suitable for the investigation of samples from FFPE tissue. However, pre-analytic parameter like time point and duration of fixation, ischemia time etc. may heavily influence the results of tissue based analyses. Assuring and reporting quality of biospecimen which are used for research has become an important matter of debate [51], [52]. According to the standard operation procedures in our department [53], the processing of oesophageal carcinoma specimens is standardized with only minimal time intervals between resection and formalin fixation. Therefore we consider our sample collection as highly homogenous in this regard. Moreover, we investigated untreated, primary resected tumours to exclude any influence on gene or protein expression which may be caused by cytotoxic preoperative treatment.

The most striking result from our study was that the prognostic protein expression pattern could only be detected by RPPA and did not correlate with mRNA gene expression analysis or immunohistochemistry (with the exception of a concordant trend for HSP27). However, some correlation was found between immunoreactivity and mRNA expression for HSP60 and HSP90. While it is well known that proteomic and genomic information often fail to correlate due to different kinetics of post-translational modifications or protein turnover [54], the predominant lack of correlation between immunohistochemistry and RPPA detected here was unexpected. This absence of correlation may be due to the higher sensitivity of RPPA as compared to the semiquantitative immunohistochemical staining. Moreover, expression levels as measured by RPPA include protein expression in stroma cells, which are present even in microdissected tumour samples. RPPA technology may deliver more subtle and accurate proteomic information as compared to immunohistochemistry and therefore may serve as an additional tool to characterize protein profiles of neoplastic and non-neoplastic cells and tissues. A possible bias concerning the interpretation of immunohistochemical analysis of molecular markers, however, could be marked intratumoral heterogeneity. This may limit the usage of tissue microarray technology in this context. We have addressed this problem in a recently published work, where we could not demonstrate a significant difference between full slide sections and TMA cores for the estimation of the expression levels of heat-shock proteins in gastrointestinal carcinomas [55]. We therefore conclude that intratumoral heterogeneity may not be the cause for the lack of correlation between PPPA and IHC. Furthermore we were not able to exactly reproduce the results of a preceding study about GRP78 and GRP94 gen-expression in the present work. This reason for this may be the application of a different methodical approach of RNA analysis reflecting the development of technical advances in this field. Moreover, when analysing single protein expression levels in our study, it was not possible to detect a relevant correlation between the clinical behaviour of the tumours in contrast to the combined expression patterns of p-HSP27^(Ser15, Ser78, Ser82)^/GRP78/GRP94/HSP60. Thus, these findings demonstrate the superiority of the identification of expression patterns of several molecular markers over the analysis of single molecules.

In summary, we detected two distinct, biologically relevant expression patterns of HSPs/GRPs in oesophageal adenocarcinomas by RPPA which was the best “biomarker” for prognosis besides lymph node status. The identification of this specific protein expression pattern in patients with a more aggressive clinical behaviour not only enhances the understanding regarding the pathogenesis of this cancer, but it might also lead the way to new specific therapy strategies directed against the inhibition of HSPs and GRPs, which have been shown to act as powerful anti-tumour agents both alone and in combination with conventional therapies.

## Supporting Information

File S1 **IHC staining pattern (orig. magnification 200x) in oesophageal adenocarcinoma samples.** Examples of strong staining reactions are shown. (A) Haematoxylin&Eosin, (B) HSP27, (C) HSP60, (D) GRP78, (E) GRP94, (F) HSP70.
(TIF)

File S2 **Quantitative protein expression levels (median and range) of HSPs and GRPs including the phosphorylated forms of HSP27 as determined by RPPA analysis and pathological parameters.**
(DOC)

File S3 **Immunohistochemical scoring of HSPs and GRPs and pathological parameters.**
(DOC)

File S4 **Relative mRNA expression levels (median and range) of HSPs and GRPs and pathological parameters.**
(DOC)

File S5 **Correlation between RPPA analysis, qPCR and immunohistochemistry.**
(DOC)
