# Supplementary figures and images for: Evidence of Prognostic Relevant Expression Profiles of Heat-Shock Proteins and Glucose-Regulated Proteins in Oesophageal Adenocarcinomas
Source: PLoS One. 2012 Jul 24;7(7):e41420. doi: 10.1371/journal.pone.0041420 (PMC3404067; doi:10.1371/journal.pone.0041420)

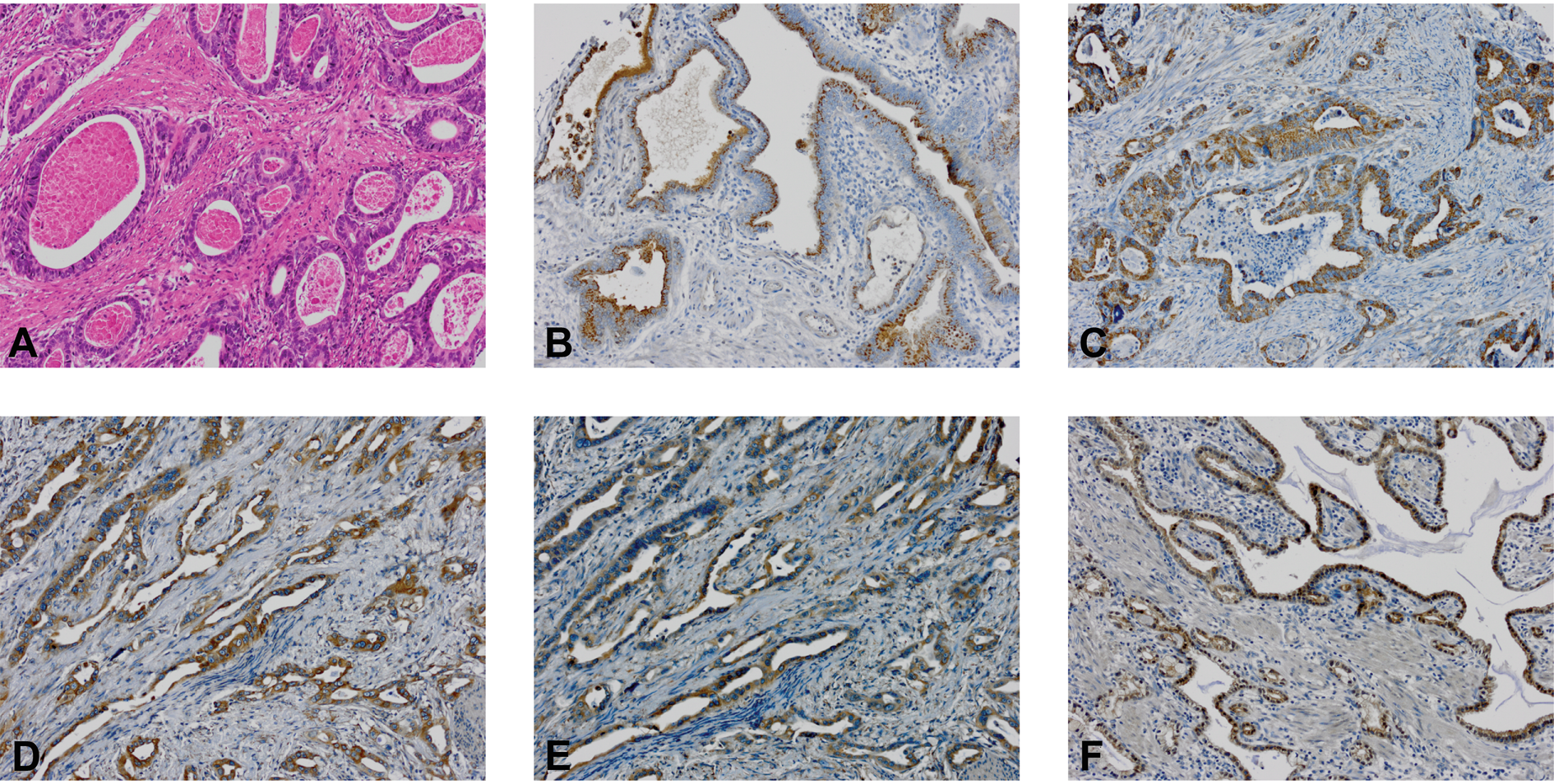

Supplement: File S1 — IHC staining pattern (orig. magnification 200x) in oesophageal adenocarcinoma samples. Examples of strong staining reactions are shown. (A) Haematoxylin&Eosin, (B) HSP27, (C) HSP60, (D) GRP78, (E) GRP94, (F) HSP70. (TIF) [file pone.0041420.s001.tif]
