# Supplementary material for: Evidence of Prognostic Relevant Expression Profiles of Heat-Shock Proteins and Glucose-Regulated Proteins in Oesophageal Adenocarcinomas
Source: PLoS One. 2012 Jul 24;7(7):e41420. doi: 10.1371/journal.pone.0041420 (PMC3404067; doi:10.1371/journal.pone.0041420)
Supplement: File S2 — Quantitative protein expression levels (median and range) of HSPs and GRPs including the phosphorylated forms of HSP27 as determined by RPPA analysis and pathological parameters. (DOC) [file pone.0041420.s002.doc]

Quantitative protein expression levels (median and range) of HSPs and GRPs including the phoshorylated forms (p-) of HSP27 as analysed by RPPA analysis and correlation with pathological parameters.

|  |  | **Median quantitative protein expression levels (min-max)** | | | | | | | | |
| --- | --- | --- | --- | --- | --- | --- | --- | --- | --- | --- |
|  |  | **HSP90** | **HSP70** | **HSP60** | **HSP27** | **p-HSP27 ^(Ser15)^** | **p-HSP27^(Ser78)^** | **p-HSP27^(Ser82)^** | **GRP78** | **GRP94** |
| ***UICC pT category*** | |  |  |  |  |  |  |  |  |  |
| pT1 | n=29 | 776  (219-3130) | 607  (167-1745) | 741  (148-2663) | 800  (0-3324) | 784  (185-2976) | 855  (215-3664) | 788  (201-4307) | 741  (303-1916) | 689  (313-2139) |
| pT2/3 | n=58 | 863  (206-2488) | 801  (151-5292) | 895  (112-3232) | 717  (0-6124) | 821  (215-3246) | 883  (245-3671) | 826  (0-2556) | 888  (174-3240) | 842  (0-4222) |
|  |  |  |  |  |  |  |  |  |  |  |
| ***UICC pN category*** | |  |  |  |  |  |  |  |  |  |
| pN0 | n=44 | 259  (219-2157) | 718  (151-2096) | 709  (148-2663) | 250  (0-4119) | 837  (185-3246) | 870  (215-3671) | 826  (201-2738) | 711  (179-1916) | 236  (0-2139) |
| pN1/2 | n=43 | 894  (206-3130) | 833  (163-5292) | 907  (112-3232) | 722  (0-6127) | 763  (215-3035) | 884  (271-3664) | 754  (0-4307) | 948  (174-3240) | 936  (254-4222) |
|  |  |  |  |  |  |  |  |  |  |  |
| ***Metastases*** | |  |  |  |  |  |  |  |  |  |
| cM0 | n=79 | 796  (206-3130) | 719  (151-5292) | 832  (148-3232) | 725  (0-6127) | 815  (185-3246) | 876  (215-3671) | 826  (0-4307) | 853  (179-3240) | 827  (0-4222) |
| cM1 | n=8 | 765  (284-1271) | 857  (271-1668) | 1140  (112-2583) | 340  (0-1017) | 662  (282-1214) | 927  (294-1103) | 656  (277-974) | 696  (174-1718) | 628  (308-3299) |
|  |  |  |  |  |  |  |  |  |  |  |
| ***Tumour grading*** | |  |  |  |  |  |  |  |  |  |
| G1/2 | n=42 | 877  (284-3130) | 691  (167-5291) | 884  (112-2970) | 636  (207-6127) | 763  (215-3136) | 863  (294-3671) | 832  (239-3015) | 829  (174-2393) | 735  (0-2195) |
| G3 | n=45 | 782  (206-2543) | 773  (151-2808) | 823  (148-3232) | 813  (0-4164) | 818  (185-3246) | 891  (215-3664) | 789  (0-4307) | 857  (179-3240) | 892  (211-4222) |
|  |  |  |  |  |  |  |  |  |  |  |
