# Supplementary material for: Evidence of Prognostic Relevant Expression Profiles of Heat-Shock Proteins and Glucose-Regulated Proteins in Oesophageal Adenocarcinomas
Source: PLoS One. 2012 Jul 24;7(7):e41420. doi: 10.1371/journal.pone.0041420 (PMC3404067; doi:10.1371/journal.pone.0041420)
Supplement: File S3 — Immunohistochemical scoring of HSPs and GRPs and pathological parameters. (DOC) [file pone.0041420.s003.doc]

Immunohistochemical stainings for HSPs and pathological parameters

|  |  | **mean Immunoreactivity Score (min-max)** | | | | |
| --- | --- | --- | --- | --- | --- | --- |
|  |  | **HSP90** | **HSP70** | **HSP60** | **HSP27** | **pHSP27^Ser15^** |
| ***UICC pT category*** | |  |  |  |  |  |
| pT1 | n=30 | 2 (0-9) | 0 (0-6) | 4 (0-8) | 2 (0-8) | 0 (0-8) |
| pT2-4 | n=62 | 2 (0-12) | 0 (0-8) | 4 (0-8) | 1 (0-8) | 0 (0-6) |
|  |  |  |  |  |  |  |
| ***UICC pN category*** | |  |  |  |  |  |
| pN0 | n=46 | 2 (0-9) | 0 (0-6) | 4 (0-8) | 2 (0-8) | 0 (0-8) |
| pN1/2 | n=46 | 2 (0-12) | 0 (0-8) | 4 (2-8) | 1 (0-8) | 0 (0-6) |
|  |  |  |  |  |  |  |
| ***Metastases*** | |  |  |  |  |  |
| cM0 | n=84 | 2 (0-12) | 0 (0-8) | 4 (0-8) | 2 (0-8) | 0 (0-8) |
| cM1 | n=8 | 2 (0-12) | 0 (0-6) | 6 (3-8) | 1 (0-6) | 0 (0-2) |
|  |  |  |  |  |  |  |
| ***Tumour grading*** | |  |  |  |  |  |
| G1/2 | n=41 | 3 (0-12) | 0 (0-4) | 4 (0-8) | 4 (1-8) | 0 (0-8) |
| G3 | n=51 | 2 (0-9) | 0 (0-8) | 4 (0-8) | 2 (0-8) | 0 (0-4) |
