# Supplementary material for: Evidence of Prognostic Relevant Expression Profiles of Heat-Shock Proteins and Glucose-Regulated Proteins in Oesophageal Adenocarcinomas
Source: PLoS One. 2012 Jul 24;7(7):e41420. doi: 10.1371/journal.pone.0041420 (PMC3404067; doi:10.1371/journal.pone.0041420)
Supplement: File S4 — Relative mRNA expression levels (median and range) of HSPs and GRPs and pathological parameters. (DOC) [file pone.0041420.s004.doc]

Relative mRNA expression levels (median and range) of HSPs and GRPs and pathological parameters

|  |  | **Median relative mRNA expression levels (min-max)** | | | | | |
| --- | --- | --- | --- | --- | --- | --- | --- |
|  |  | **HSP90** | **HSP70** | **HSP60** | **HSP27** | **GRP78** | **GRP94** |
| ***UICC pT category*** | |  |  |  |  |  |  |
| pT1 | n=30 | 785  (220-2250) | 1085  (300-2500) | 2270  (650-7890) | 40  (10-160) | 491  (160-1250) | 1080  (470-2210) |
| pT2/3 | n=62 | 835  (250-5111) | 1256  (440-4519) | 3060  (870-11430) | 70  (10-220) | 484  (90-1660) | 1130  (440-3780) |
|  |  |  |  |  |  |  |  |
| ***UICC pN category*** | |  |  |  |  |  |  |
| pN0 | n=46 | 790  (220-5110) | 1130  (300-2540) | 2260  (650-7890) | 50  (10-180) | 491  (140-1250) | 1080  (470-2210) |
| pN1/2 | n=46 | 790  (250-4600) | 1210  (440-4510) | 3030  (900-11143) | 55  (10-220) | 490  (90-1660) | 1180  (440-3780) |
|  |  |  |  |  |  |  |  |
| ***Metastases*** | |  |  |  |  |  |  |
| cM0 | n=84 | 800  (220-5110) | 1210  (300-4510) | 2535  (650-11430) | 60  (10-220) | 491  (90-1660) | 1090  (440-3780) |
| cM1 | n=8 | 630  (380-2910) | 1200  (440-2260) | 2510  (1470-3970) | 60  (10-120) | 417  (180-940) | 810  (580-1700) |
|  |  |  |  |  |  |  |  |
| ***Tumour grading*** | |  |  |  |  |  |  |
| G1/2 | n=41 | 760  (250-5110) | 1410  (440-3060) | 2380  (650-7890) | 60  (10-210) | 549  (140-1250) | 1080  (440-2210) |
| G3 | n=51 | 800  (220-2870) | 1140  (300-4510) | 2660  (820-11430) | 60  (10-220) | 472  (90-1660) | 1070  (470-3780) |
|  |  |  |  |  |  |  |  |
