# Supplementary material for: Evidence of Prognostic Relevant Expression Profiles of Heat-Shock Proteins and Glucose-Regulated Proteins in Oesophageal Adenocarcinomas
Source: PLoS One. 2012 Jul 24;7(7):e41420. doi: 10.1371/journal.pone.0041420 (PMC3404067; doi:10.1371/journal.pone.0041420)
Supplement: File S5 — Correlation between RPPA analysis, qPCR and immunohistochemistry. (DOC) [file pone.0041420.s005.doc]

Correlations between RPPA analysis, qPCR and immunohistochemistry (IHC)

|  | **Correlation (correlation coefficient r, p-value)** | | |
| --- | --- | --- | --- |
|  | RPPA/ IHC | RPPA/qPCR | IHC/ qPCR |
| **HSP27** | r=0.054; p=0.611 | **r=0.272, p=0.010** | r=-0.141, p=0.189 |
| **Phospho-HSP^(Ser15)^** | r=0.126; p=0.231 | r=0.104; p=0.329 | r=0-.062; p=0.564 |
| **Phospho-HSP^(Ser78)^** | r=-0.077; p=0.466 | r=0.145, p=0,173 | r=0.086; p=0.422 |
| **Phospho-HSP^(Ser82)^** | r=-0.003; p=0.978 | r=0.046; p=0.665 | r=0.176; p=0.099 |
| **HSP60** | r=-0.068; p=0.519 | r=-0.156; p=0.141 | **r=0.246; p=0.020** |
| **HSP70** | r=0.060; p=0.567 | r=-0.154; p=0.146 | r=0.037; p=0.726 |
| **HSP90** | r=-0.081; p=0.443 | r=-0.065; p=0.539 | **r=0.306; p=0.003** |
| **GRP78** | r=-0.21; p=0.858 | r=-0.087; p=0.413 | r=0.002: p=0.987 |
| **GRP94** | r=-0.111; p=0.335 | r=-0.174; p=0.099 | r=0.046; p=0.690 |
